# Delivery of affordable and scalable encapsulated allogenic/autologous mesenchymal stem cells in coagulated platelet poor plasma for dental pulp regeneration

**DOI:** 10.1038/s41598-021-02118-0

**Published:** 2022-01-10

**Authors:** Ioannis Angelopoulos, Cesar Trigo, Maria-Ignacia Ortuzar, Jimena Cuenca, Claudia Brizuela, Maroun Khoury

**Affiliations:** 1 Laboratory of Nano-Regenerative Medicine, Faculty of Medicine, Universidad de Los Andes, Santiago, Chile; 2 Cells for Cells and REGENERO, The Chilean Consortium for Regenerative Medicine, Santiago, Chile; 3 Centro de Investigacion en Biologia y Regeneracion Oral (CIBRO), Faculty of Dentistry, Universidad de los Andes, Santiago, Chile; 4 IMPACT, Center of Interventional Medicine for Precision and Advanced Cellular Therapy, Santiago, Chile

**Keywords:** Regeneration, Biomaterials - cells

## Abstract

The main goal of regenerative endodontics procedures (REPs) is to revitalize teeth by the regeneration of healthy dental pulp. In this study, we evaluated the potential of combining a natural and accessible biomaterial based on Platelet Poor Plasma (PPP) as a support for dental pulp stem cells (DPSC) and umbilical cord mesenchymal stem cells (UC-MSC). A comparison study between the two cell sources revealed compatibility with the PPP based scaffold with differences noted in the proliferation and angiogenic properties in vitro. Additionally, the release of growth factors including VEGF, HGF and DMP-1, was detected in the media of cultured PPP and was enhanced by the presence of the encapsulated MSCs. Dentin-Discs from human molars were filled with PPP alone or with MSCs and implanted subcutaneously for 4 weeks in mice. Histological analysis of the MSC-PPP implants revealed a newly formed dentin-like structure evidenced by the expression of Dentin sialophosphoprotein (DSPP). Finally, DPSC induced more vessel formation around the dental discs. This study provides evidence of a cost-effective, xenofree scaffold that is compatible with either autologous or allogenic strategy for dental pulp regeneration. This attempt if successfully implemented, could make REPs treatment widely accessible, contributing in improving global health conditions.

## Introduction

Endodontic therapy is one of the most used techniques in dental practice with a prevalence that varies between 58.7 and 78%, depending on the population studied^1,2^.Regenerative endodontic procedures (REPs) has been defined as biology based procedures designed for resolving the disease and restoring of some or all of the original tissues function that have been damaged^3^. Dental pulp is responsible to provide nutrition to the tooth and protection from harmful pathogens. Infected dental pulp due to dental caries leads evidently to an invasive root canal therapy where the removal of the dental pulp is necessary in order to stop the infection and eventually its replacement with non-biodegradable material such as gutta-percha^4,5^. Patients that have received a root canal therapy often face problems with vitality of the dental pulp in the long term and more importantly an increase in the tooth fragility making it susceptible to fractures and eventually the necessity of a complete tooth replacement with a titanium implant^6–11^. There is a need for other clinical options based on novel regenerative endodontics strategies. These advanced therapies should be able of the generation of new tissues with the characteristics and function similar of healthy dental pulp vascularization, re-innervation, and dentin deposition^12,13^. Despite the fact that the dental conditions targeted by REPs are more prevalent in developing than developed countries the procedure to be approved in the future would be inaccessible to a large part of the population. The additional challenge for regenerative medicine (RM) in general and ER is to bring manageable and cost-effective treatment to a large part of the world, contributing in improving global health conditions. In the study of the World Morbidity Burden of 2010, it was determined that the market for oral pathologies maintains a high prevalence, affecting altogether 3900 million people. Untreated tooth decay was the most prevalent condition affecting a total of 2431 million individuals, of the world population^14^. In order to achieve functional pulp regeneration usually the need of three features are essential such as (1) stem cells, (2) scaffolds and (3) signaling molecules^15^. Among the first efforts to regenerate the dental pulp was the work from Moony and Rutherford were they work on the isolation and characterization of DPSC and their potential differentiation^16^, while other laboratories have worked on DPSC differentiation towards odontoblast-type cells^17,18^. Mesenchymal stem cell (MSC) transplantation (autologous or allogenic) could offer an alternative solution for dental pulp regeneration. MSC have been used previously in other type of cell-based therapies due to their unique properties. MSC are multipotent stem cells that can differentiate into different cell types (osteogenic, chondrogenic and adipogenic phenotypes) and they possess immunomodulatory properties and low immunogenicity, as they don’t express MHC class II and CD40, CD80 and CD86 molecules and finally express low levels of MHC class I positioning them as prominent candidates for the treatment of several conditions such graft-versus-host disease (GVHD) and autoimmune diseases^19^.Additionally, they possess self-renewal properties and could secrete angiogenic and other growth factors^20–22^.The main advantage of autologous cell-based therapies is being immunologically matched, hence, the lack of immune rejection associated with donor graft eliminates the need to administer costly immunosuppressive drugs^23–26^. However, autologous therapies require point-of-care facilities to manufacture cell-based products at the bedside. Another limitation is that the cells are usually harvested from older patients, which can greatly hinder their biological activities and in consequence the therapeutical outcome^19^. Consequently, these disadvantages and logistic burdens signify difficulties for standardization and up scaling, therefore, limiting their commercialization widespread. On the other hand, allogeneic MSC sourced mainly from young healthy donors could represent a good alternative, with the benefit of being an off-the-shelf, ready-to-inject product making them a potential business model. Additionally, the potential to be scaled-up, the improved quality control and batch standardization makes them appealing for commercial perspectives. On the other hand, as the transplanted allogeneic cells can differentiate in different lineages, therefore, by losing their stem characteristics, they can begin to express antigens that are recognized by the immune system. The consequence of losing their immunoprivilege status results in limiting their long-term paracrine and tissue integration effects. Taking into consideration of the pros and cons of Allo-MSC transplantation, there have been several clinical trials using allogenic bone marrow-derived MSCs without producing any clinically relevant immune reactions^27–32^. Specifically, It has been reported that allo-MSCs inhibit the activity of various immune cells, including T cells, B cells, natural killer cells, and dendritic cells. These immunosuppressive properties in combination with low immunogenicity results to reduced immune response after implantation^19^. The combination of cell delivery and biomaterials that could be used as vehicle to deliver agents to the site of infection/injury including regeneration will overcome some of the hurdles and could offer a great hope for the dental regeneration field^33^. Previously, different biomaterials with the combination of cells such fibroblasts isolated from the dental pulp seeded on a syntetic Polyglycolic acid (PGA) scaffold and after 60 days of culture was demonstrated that are capable of forming new dentin tissue^16^.This huge potential of regenerative medicine (RM) comes at a substantially important cost. Even though the high-price can be justified for life-saving treatment, it is difficult to vindicate it for dental repair. While the choice of sophisticated synthetic biomaterials, new manufacturing technique and routes of their implantation might improve the clinical outcomes for tissue regeneration, however, these developed technologies make the final treatment largely unaffordable. There is a clear need for natural, low-cost material and procedures for RE. Plasma Poor Platelet (PPP) is a blood derivative very similar to Platelet Rich Plasma (PRP) but with a reduced number of platelets. The use of platelet concentrates is recent, but its efficiency remains controversial. Several techniques for platelet concentrates are available, mostly using the patient’s own plasma; however, their applications have been confusing leading to inconsistent outcome. This is largely due to donor variability, and lack of a proper procedure standardization^34^. In a previous study, a comparison of the growth factor release profile between PRP and PPP concludes that both PRP and PPP have the same growth factors profile and both provide a similar stimulus for bone differentiation^35^. PRP has been used in wound healing to facilitate migration and proliferation of fibroblasts^36^ and also for bone defects^37,38^. Additionally, PRP has been used for different endodontic procedures instead of a blood clot for regeneration purposes, taking advantage of its rich content with adhesive glycoproteins, which can react with cell receptors and facilitate cell attachment to the scaffold and also provides a reduced risk of an immunological reaction when used as a scaffold^39^. In this work, we evaluated the potential of combining all 3 fundamental elements of tissue engineering: (1) stem cells (SCs), (2) cytokines/growth factors, and (3) microenvironment (scaffold). For this, we follow 3 main selection criteria for these elements, including the exclusive use of xeno-free, non-synthetic and low-cost materials. The main objectives of this study was firstly, to compare the biological properties such as migration, proliferation and angiogenesis between autologous DPSC and allogeneic UC-MSC and secondly, to investigate the efficacy of PPP as a cell delivery agent of allogenic or autologous MSC using a dentin/disc mouse model for dental pulp regeneration. This combination of affordable carrier with scalable cell production provides a manageable and cost-effective approach for dental pulp regeneration. This attempt if successfully implemented, could bring affordable RE treatment to a large part of the world, contributing in improving global health conditions.

## Material and methods

### Isolation of DPCS and UC-MSC in vitro expansion

Molars were extracted from three healthy individuals (one man and two women) aged between 18 and 25 without any evidence of dental caries, in the dental school of Universidad de Los Andes, San Bernardo. After signing an informed consent form and following the ethical approval of the Universidad de los Andes as described previously^40^. Umbilical cords were isolated from full-term human placentas of male newborns collected from cesarean deliveries after informed consent from the maternal donors, and ethical revision and approval from the ethics committees of both the Clínica Davila Hospital and the “Servicio de Salud Metropolitano Oriente” in Chile as described previously^41^. The dental pulp stem cells (DPSC) and the umbilical cord stem cells (UC-MSC) were isolated with direct cell outgrowth from the tissue explants. Both explants were incubated for 20 days until the dish reach confluence and MSC with a fibroblast-like morphology were observed^42^. All experiments were performed in accordance with relevant guidelines and regulations of Universidad de los Andes.

### Immunophenotypical profile by flow cytometry and mesodermal differentiation

For the immunophenotypic characterization, DPSC or UC-MSC were incubated with the following antibodies: CD105, CD90, CD73, CD34, CD45,CD19 and HLA-DR (BD, USA) for 20 min at 4 °C in dark area, then were washed with 4 ml of PBS 1X, centrifuged at 1800 rpm for 6 min and the supernatant was removed. Data were collected using a FACS Canto II Flow cytometer (BD Biosciences, San Jose, CA) and analyzed with FlowJo analysis software.The protocol for mesodermal differentiation (Adipogenic, Osteogenic, Chondrogenic) was performed as described previously^40,42^.

### Cell Proliferation (alamarBlue™) and cell migration assay

To compare the proliferation capacity between DPSC and UC-MSC, 1000 cells were plated in a 24 well (Nunc, USA) with proliferation medium α-MEM (10% FBS and 1% Penn Strep) at 37 °C, 5% CO_2_. The proliferation rate was measured at various time points (day 1, 3, and 9) using the alamarBlue™ (Invitrogen, USA). The fluorescence intensity was measured using a plate reader (Tecan, USA) at 570 nm. The cell migration was evaluated with an in vitro scratch assay as described previously^40^. Cells (350.000) were seeded in a 6 well plate with proliferation α-MEM (10% FBS and 1% Penn Strep) at 37 °C, 5% CO_2_. After 24 h of incubation, a scratch was made with a 10 μl pipet tip (Thermoscientific, USA). Images were taken at various time points (0, 4, 8, 12 and 24 h) using an inverted microscope until the complete closure of the gap. The images were analyzed with the Wimscratch Software (Wimasis, Germany).

### In vitro and in vivo angiogenic comparison of DPCS and UC-MSC and measurement of angiogenic factors

The angiogenic potential of DPSC or UC-MSC*in vitro* was evaluated based on their capacity to form tube-like structures in vitro (Total Branching Points, Total tube Length, Total Loops). Also, to determine the angiogenic potential of MSC conditioned media (CM). DPSC or UC-MSC were incubated under hypoxic (1% O_2_) or normoxic conditions for 48 h and subsequently, the MSC-CM was incubated with HUVEC (human umbilical vein endothelial cells) coated with Matrigel Matrix (EGM-2 and α-MEM were used as control). Additionally, the different MSC-CM was collected and the secreted levels of VEGF and HGF were measured using the DuoSet ELISA Development System (R&D Systems, USA). Finally, to compare the angiogenic potential of DPSC and UC-MSC in vivo, the Matrigel plug assay was performed in an 8-week-old NOD.Cg-Prkdc^scid^ Il2rg^tm1Wjl^/SzJ (NSG) mice (Jackson Laboratories, Bar Harbor, ME). All in vivo studies received approval by the Universidad de Los Andes ethical committee for animal experimentation and have been followed the in vivo experiments (ARRIVE) guidelines/checklist for preclinical animal studies. The experimental procedure has been described previously^40^.

### Platelets poor plasma (PPP) scaffold fabrication and in vitro biocompatibility of DPSC and UC-MSC

PPP from AB Rhesus positive, as universal plasma donors (500 ml) was obtained from the blood bank unit of the *Clínica Universidad de los Andes*, Santiago-Chile. To avoid several freeze–thaw cycles, PPP was aliquoted it in falcon tubes (50 ml) and maintained at − 80 °C until further used. To prepare a PPP scaffold (5 ml of PPP total), 1 × 10^6^ cells were mixed with 3800 μl of PPP, 875 μl α-MEM (10% FBS and 1% Penn Strep), 75 μl oftranexamic acid and 250 μl of 2% Calcium chloride and transferred in a 6 well plate (Nunc, USA) and placed at 37 °C until further use. The in vitro biocompatibility was evaluated with alamarblue™ (Invitrogen, USA) after 1, 3, 7 and 14 days. Several puncheswere generated in the PPP using a 5 mm diameter punch (Dolphin Medical, Chile). Each punch was placed separately in a 96 well plate and covered with 200 μl of culture medium and20μl of alamarBlue™ reagent and incubated for 2 h at 37 °C. After the incubation period 100μlof the supernatant were transferred in a fresh 96 well plate and the absorbance was measured according to the manufacturer instructions. In parallel, punches of PPP scaffolds for each time point (1, 3, 7 and 14 days) were fixed in 10% formalin solution and processed for histological analysis. The scaffolds were stained for hematoxylin and eosin (Sigma, USA).

### Ultra-structural analysis of PPP scaffolds and porosity

To evaluate the ultra-structural analysis, at different time points, Scanning Electron Microscopy (SEM) was used to investigate the structure, morphology of the PPP scaffolds with or without cells. The 5 mm punches of PPP scaffolds described previously were fixed with 2.5% glutaraldehyde (Sigma, USA), dehydrated in a progressive series of ethanol before being mounted on an aluminum stub using silver paint. Samples were coated with gold/palladium before examination under a JSM‐7500F scanning electron microscope (JEOL, USA). PPP scaffold pore size quantification: SEM images were subjected to binarization by the “threshold” function of the Image J software. In the binary images, black pixels represent the porosity content of the PPP. Then the percentage of black pixels was measure in twenty box regions of 400 µm^2^ per image which represents the mean porosity content.

### Measurement of released protein factors from PPP

An ELISA was performed to evaluate the protein content and release profile of PPP with or without the encapsulated cells (DPSC/UC-MSC). PPP scaffolds were processed as described above. At 24 h post- incubation, the conditioned medium (CM) was collected and the PPP was carefully detached, lifted from the 6 well plate and finally squeezed using a 10 ml syringe (BD, USA) with a 16G needle (Hamilton, USA) until all the exudate of the PPP was completely removed. The two different liquids (CM + Exudate) were processed and the secreted levels of VEGF, HGF and DMP-1 were measured using the DuoSet ELISA Development System (R&D Systems, USA).

### Preparation of dentin-discs/PPP scaffolds and implantation into a mouse subcutaneously

Human molar teeth extracted from healthy individuals as described previously were sliced horizontally with thickness of 3 mm and length 0.5 mm using a Dremel saw (100-N/6 220-Volt Single Speed Rotary Tool Kit). The volume of the root canal space was 40 μl. To avoid microbial contamination, the dentin-discs were sterilized using a 17% ethylene-diamine-tetra-acetic acid solution for 10 min and 19% citric acid for 1 min to remove the smear layer, followed by treatment with betadine for 30 min and 5.25% NaOCl for 10–15 min. Finally, discs were rinsed in sterile PBS and incubated at 37 °C for 3–7 days. Cells (1 × 10^6^) were mixed with 760 μl of PPP, 175 μl α-MEM (10% FBS and 1% Penn Strep), 15 μl of tranexamic acid and 50 μl of 2% Calcium chloride in a 1.5 centrifuge tube. Upon coagulation, PPP was detached and lifted carefully using a pair of tweezers and placed into the canal space of each root a fragment and kept in a 6 well plate with proliferation medium (α-MEM) at 37 °C until further use. Two dentin/PPP scaffolds were implanted subcutaneously on the dorsum of a 6–8 weeks old NOD.Cg-Prkdcscid Il2rgtm1Wjl/SzJ (NSG) mouse (Jackson Laboratories, Bar Harbor, ME) for a period of 30 days. The mice (18 mice in total) were divided into 3 different groups: (1) PPP + dentin (2) PPP + DPSC + dentin and (3) PPP + UC-MSC + dentin. The mice were euthanized using CO_2_and images were taken of the implant discs while still positioned on the mouse skin to quantify new vessels formed around Dentin-Discs/PPP Scaffolds using image J as described previously. Thereafter, the implants were removed and placed in 10% formalin (Sigma, USA) and then decalcified for 2 months using formic acid (Sigma, USA). Finally, they were paraffin embedded and longitudinally sectioned (4 μm sections) and stained for hematoxylin and Eosin (Sigma, USA). Some of the sections were used for immunohistochemical analysis. All animal procedures followed a protocol approved by the Institutional Animal Care and Use Committee at the Universidad de los Andes and have been followed the In Vivo Experiments (ARRIVE) guidelines/checklist for preclinical animal studies.

### Immunohistochemistry

For immunohistochemistry, deparaffinized sections were dehydrated in a series of xylol and alcohol series and then the antigen recuperation was performed using citric buffer. The samples were immersed in 3% H_2_O_2_ for 15 min and then blocked with BSA for 30 min. After the primary incubation overnight at 4 °C was performed using the following antibodies: (1) Human leukocyte antigen (Anti- HLA-A) (ABCAM, ab52922, USA) and Dentin sialophosphoprotein (DSPP) (ABCAM, ab12232, USA). Isotype-matched control antibodies were used under the same conditions as the primary antibodies. For enzymatic immunohistochemical staining, VECTASTAIN® Universal ABC kit (Vector Laboratories, USA) according to the manufacturer’s protocol. All sections were counterstained with hematoxylin and mounted with a 10 µl drop of Entellan (MercK). The amount of protein expression was calculated using image J software and was expressed as % area.

### Statistical analysis

All the experiments were performed in biological and experimental triplicated and the values expressed as mean. The GraphPad Prism version 7 software was used for statistical analysis. The comparisons between the groups were made with one-way ANOVA post-hoc Tukey or Bonferroni tests. A probability value of *P* < 0.05 (*), *P* ≤ 0.01 (**), *P* ≤ 0.001 (***) and *P* ≤ 0.0001 (****) was considered statistically significant.

## Results

### DPSC display a higher proliferation rate in comparison with UC-MSC

**Figure 1:**
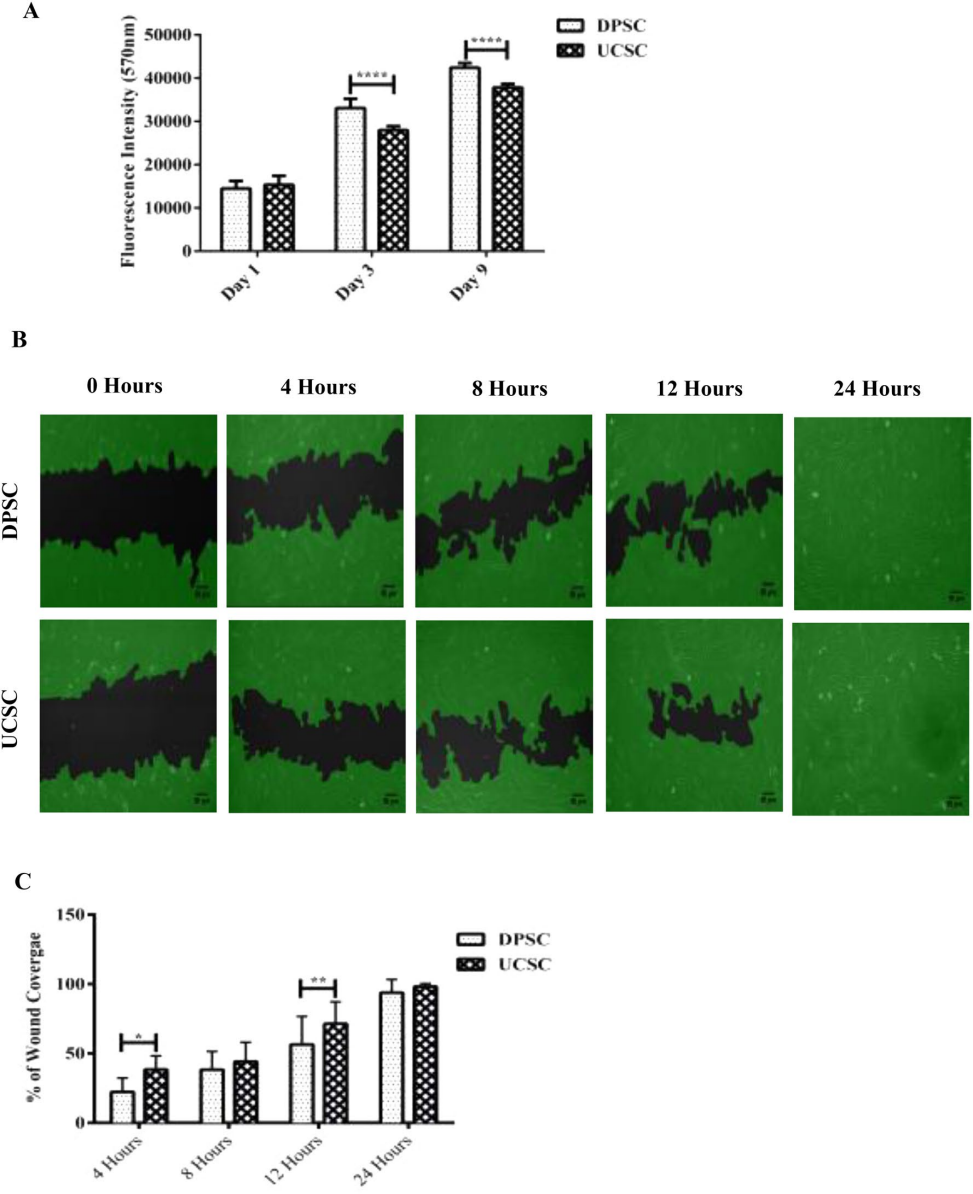
DPSC and UC-MSC showed different clonogenic proliferation potentials. Quantification of cell proliferation between DPSC and UC-MSC incubated at different time points (1, 3 and 9 days) (**A**) A significant increase in the proliferation of DPSC at day 3 and day 9 (*P* < 0.0001). (**B**) In vitro migration comparison between DPSC and UC-MSC based on a 24 h scratch wound healing assay. (**C**)The migratory capacity was evaluated from each time point (4, 8 and 12 h) in correlation to 0 h (Images not shown). There was a significant increase in the migration of UC-MSC compared to DPSC at 4 h (*P* < 0.05) and 12 h (*P* ≤ 0.01) respectively. No significant difference was observed at 8 at 24 h. All data are represented as a mean with the associated SEM (*n* = 3) of a minimal three donors.

Both cell sources showed a positive expression of the common MSC markers such as CD105, CD90, CD73, CD34, CD45,CD19 and HLA-DR (Fig. Supplementary 1A) and also DPSC and UC-MSC were induced to differentiate into mesodermal lineages (Fig. Supplementary 1B). No apparent differences were observed between DPSC or UC-MSC and also their multipotency (osteogenic, chondrogenic, adipogenic).DPSC and UC-MSC have shown previously similar fibroblast-like characteristics^43,44^. Additionally, the proliferation between the DPSC and UC-MSC was investigated using an alamarBlue™ cell proliferation assay. A significant increase in the proliferation of DPSC at day 3 and day 9with 1.2- and 1.1-fold increase respectively (*P* < 0.0001) (Fig. 1A).

### UC-MSC exhibit a superior migratory capacity in a wound scratch assay

To evaluate the migration potential of DPSC and UC-MSC, a wound scratch assay was performed. The migratory capacity was evaluated at different time points (0, 4, 8 and 12 h). There was a significant increase in the migration of UC-MSC compared to DPSC at 4 and 12 h with 1.2-fold (*P* < 0.05) and 1.4-fold (*P* ≤ 0.01) increase respectively. No significant difference was observed at 8 h and at 24 h where in the latter time point full wound closure was reached by both cell sources. This experiment indicates that UC-MSC possess a higher migration potential in comparison to DPSC for the different time points analyzed (Fig. 1B + C).

### DPSC were able to form a higher number of tube-like structures compared to UC-MSC

**Figure 2:**
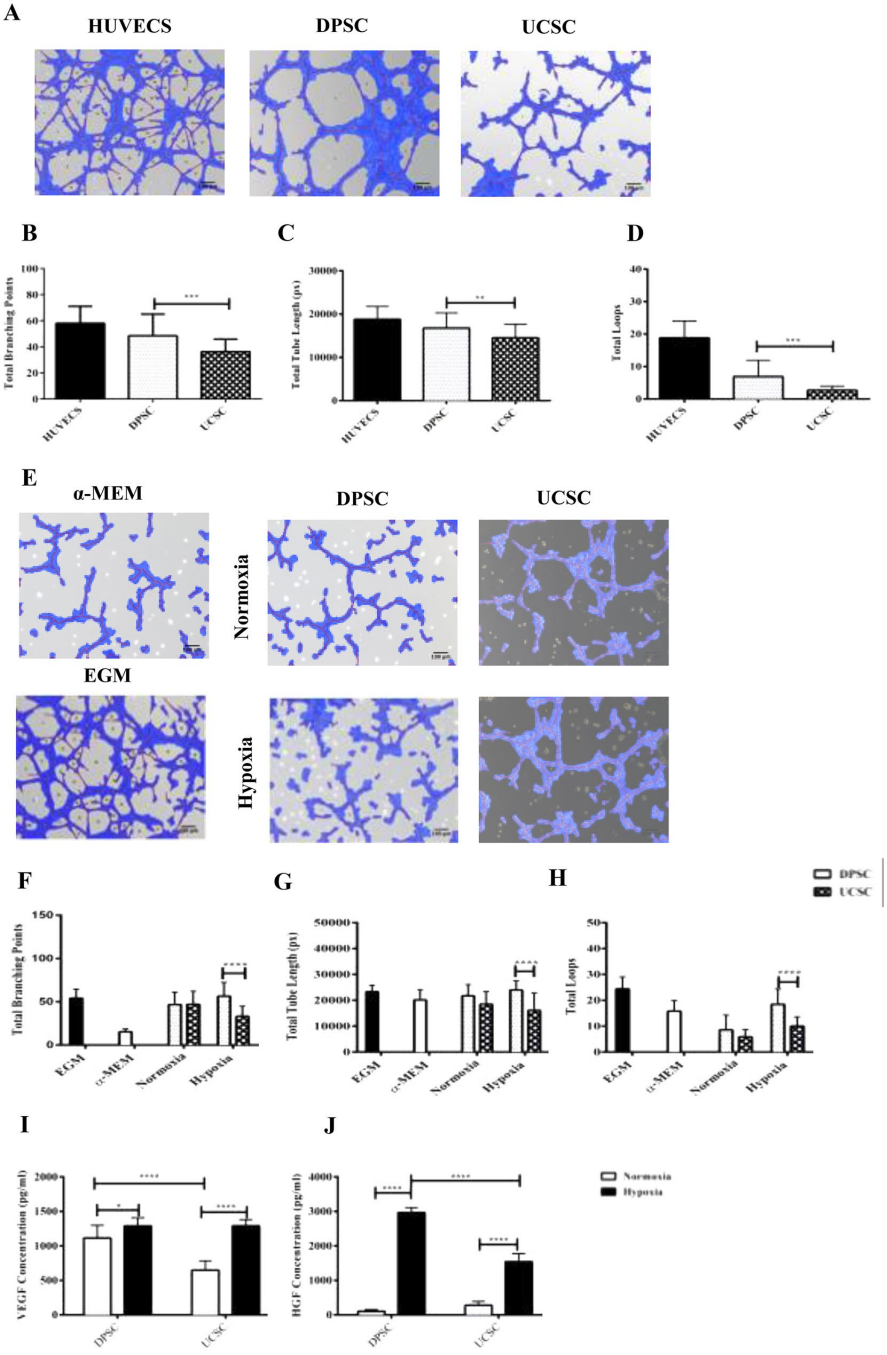
In vitro angiogenesis comparison between DPSC and UC-MSC based on a 5 h exposure to Matrigel. (**A**) Images were analyzed using a Wimasis software. (**B**) DPSC were shown a higher potential to form (**B**) branching points (*P* ≤ 0.001), (**C**) total tube length (*P* ≤ 0.01) and (**D**) total loops (*P* ≤ 0.001), in Matrigel coating cultures. HUVECS were used a control. In vitro angiogenesis comparison between DPSC and GMSC conditioned media under hypoxic and normoxic conditions. (**E**)Images were analyzed using a Wimasis software. The DPSC conditioned media under hypoxic conditions were shown a better potential to form: (**F**) total branching points (*P* ≤ 0.0001), (**G**) total tube length (*P* ≤ 0.0001)and (**H**) total loops (P ≤ 0.0001) compared to supernatant of UC-MSC. EGM and α-MEM medium were used a control.Protein levels of VEGF and HGF were measured by ELISA. (**I**) No significant difference was observed in the VEGF protein levels between hypoxic DPSC vs UC-MSC. (**J**) However, there was an increase in the HGF protein levels (*P* < 0.05) (**J**) (*P* ≤ 0.0001). All data are represented as a mean with the associated SEM (*n* = 3) of a minimal three donors.

The angiogenic ability designated by the ability of DPSC and UC-MSC to form tubular network was investigated in vitro in a semi-solid medium (Matrigel). The in vitro angiogenesis was evaluated with following characteristics: (1) total branching points, (2) total tube length and (3) total loops (Fig. 2A). Image analysis of the tube formation evaluated at 5 h post-culture initiation, showed a higher angiogenic capacity evidenced by a more extensive network of capillary-like structures for DPSC as compared to UC-MSC. Specifically, a 1.3-fold total branching points increase (*P* ≤ 0.001), 1.15-fold change total tube length (*P* ≤ 0.01) and a 2.5-fold change for total loops (*P* ≤ 0.001) (Fig. 2B + C + D). To evaluate the secreted paracrine factors, we assessed the angiogenic potential of released factors from the conditioned media (CM) harvested from DPSC and UC-MSC after 48 h incubation under hypoxic (1% O_2_) or normoxic conditions. HUVECS were re-suspended with the CM and were seeded onto pre-coated plated with growth Factor Reduced Matrigel. Alpha-MEM (basal media) and EGM (angiogenic media) were used as the negative and positive controls, respectively. The tube formation was analyzed after 5 h of incubation. Images were taken and the results have shown a higher tubular structure for the HUVECS incubated with the conditioned medium under hypoxic conditions vs normoxic (Fig. 2E). There was a significant difference between the formation of total tube lengths, total loops, and total branching points between hypoxia DPSC and hypoxia UC-MSC. Specifically, a 1.7 fold change for total branching point increase(*P* ≤ 0.0001), a1.4 fold change for total tube length(*P* ≤ 0.0001) and a 1.8 fold change for total loops(*P* ≤ 0.0001) were observed in favor of DPSC, as showed in Fig. 2F + G + H. The quantification of VEGF and HGF (Fig. 2I + J) revealed a significant increase under hypoxic conditions for both cell sources. However, only the release of HGF was 1.9-fold (*P* ≤ 0.0001) higher after 48 h of incubation for DPSC compared to UC-MSC.

### Angiogenic potential of UC-MSC and DPSC in vivo

**Figure 3:**
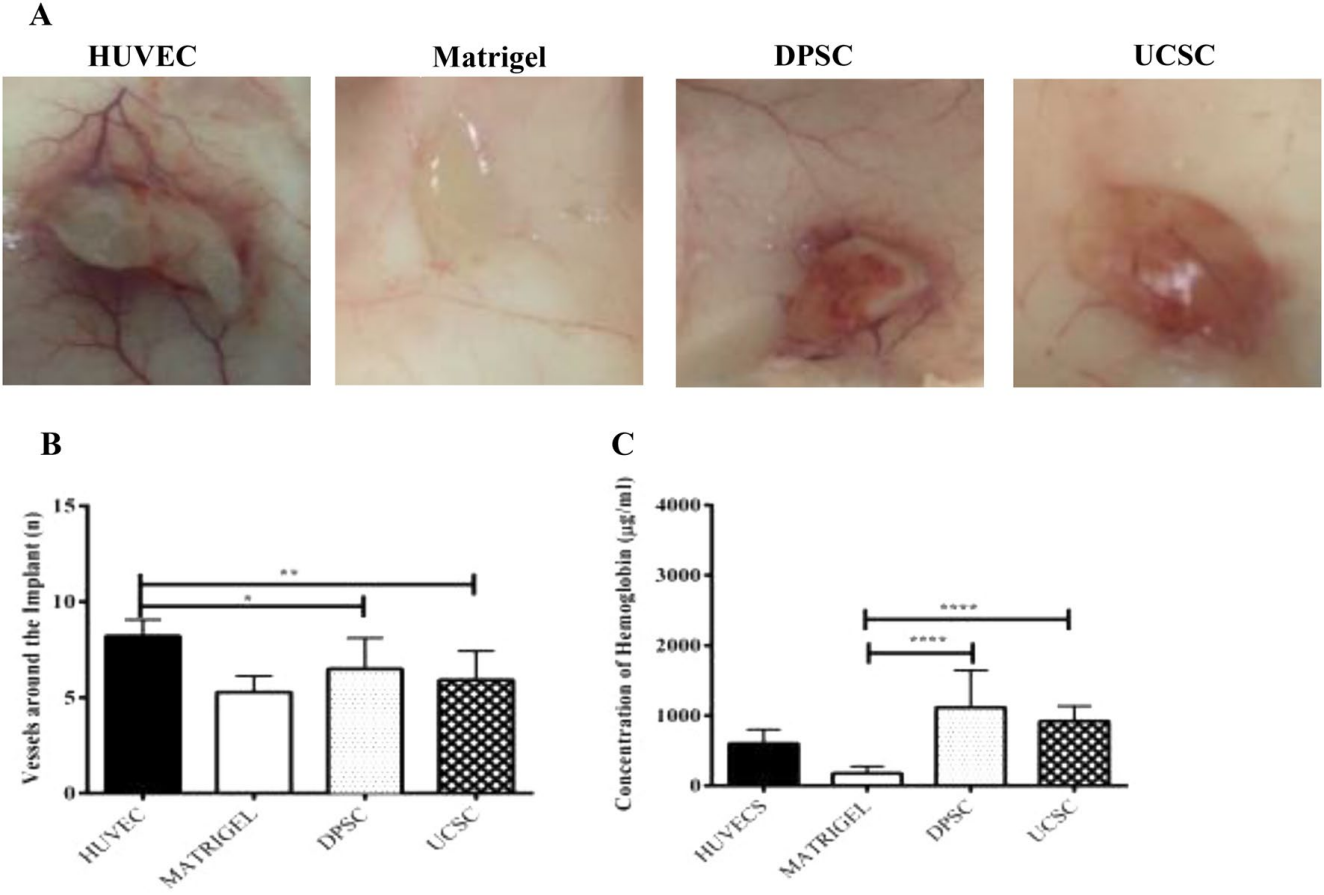
Evaluation of the angiogenic potential of DPSC and UC-MSC in a plug assay model. To determine the angiogenic capacity between DPSC and UC-MSC in vivo a Matrigel plug assay was performed in NSG mice. The mice were dived into four different groups HUVEC (positive control), Matrigel (Negative Control), DPSC and UC-MSC. The different cells (2 × 10^6^) were mixed with a growth factor reduced Matrigel and implanted subcutaneously. At 15 days post transplantation the implants were harvested and (**A**) images were taken and (**B**) Quantification of the vessels around the implant was performed using the image J software. The results have shown a non-significant difference in the vessel formation between DPSC and UC-MSC. Additionally, (**C**) a quantification of the hemoglobin content (μg/ml) was performed using Drabkin´s reagent at different concentrations. The results have shown an increase in the hemoglobin content of DPSC and UC-MSC compared to Matrigel (negative control) (*P* ≤ 0.0001) respectively.

To comparatively evaluate the angiogenic potential of DPSC and UC-MSC in vivo, cells were embedded in a Matrigel plug then implanted in a NSG mouse. After 15 days the implants were collected, and photographs were taken. As shown in Fig. 3A, all plugs generated vessels around and inside the implant, with the highest number reached by the HUVEC-plug, used as a positive control. The image analysis of the implants showed a non-significant difference in the vessel formation between DPSC and UC-MSC (Fig. 3B). Additionally, the implants were extracted and analyzed for their hemoglobin (Hb) content. The quantification of the Hb resulted in 6.2 folds and 5.1 folds higher hemoglobin content of DPSC and UC-MSC respectively when compared with a cellular plug (Matrigel alone) (*P* ≤ 0.0001) (Fig. 3C).

### Cytocompatibility of DPSC or UC-MSC encapsulated in the PPP scaffolds and ultrastractural analysis

**Figure 4:**
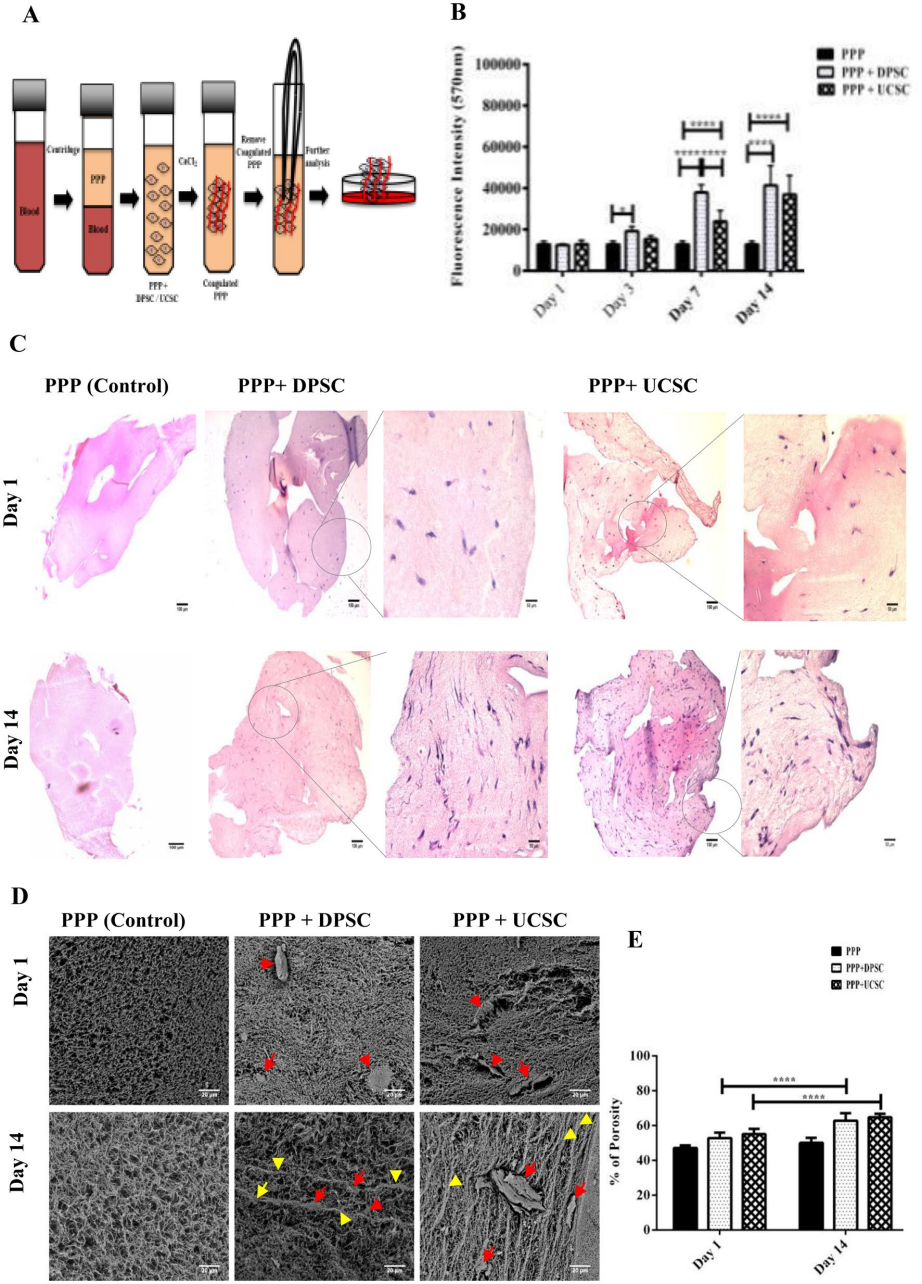
(**A**) Illustration of the preparation of the Plasma Poor in Platelets (PPP). Whole blood was centrifuged, and the PPP was collected and mixed with either DPSC or UC-MSC. Later calcium chloride at 2% was added and the PPP was coagulated. The coagulated PPP was removed with a tweezers and placed in a petri dish with α-MEM for further processing. Biocompatibility of DPSC or UC-MSC encapsulated on PPP scaffolds. **(B**) Proliferation profile of DPSC and UC-MSC encapsulated in PPP evaluated by alamarBlue™ was used to detect the staining, showing an increase in the proliferation between DPSC + PPP vs PPP alone at day 3 (*P* < 0.05), day 7 (*P* ≤ 0.0001) and day 14 (*P* ≤ 0.0001). Also a significant increase was observed between UC-MSC + PPP vs PPP alone at day 7 (*P* ≤ 0.0001) and 14 (*P* ≤ 0.0001). Finally, a significant difference was observed between UC-MSC + PPP and DPSC + PPP at day 14 (*P* ≤ 0.0001). All data are represented as a mean with the associated SEM (*n* = 3) of a minimal three donors. (**C**) Additionally, histological analysis with H&E of DPSC and UC-MSC in PPP at various time points Day 1 and Day 14. PPP without cells was used as control. Scanning Electron Microscopy (SEM) and porosity analysis of PPP. (**D**) PPP alone and PPP + Cells (DPSC or UC-MSC) were imaged using an electron microscope. Red arrowheads point at the presence of cells, yellow arrowheads point at the formation of organized fibers at Day 14 of the culture of PPP encapsulated with either DPSC or UC-MSC. The structure of the PPP alone has a random orientation at Day 1. (**E**) Pore sizes were quantified using image J software. There was an increase in the pore size after 14 days of incubation in either UC-MSC + PPP or DPSC + PPP compared to Day 1 (*P* ≤ 0.0001).

To evaluate the compatibility of the selected natural scaffold, UC-MSC or DPSC were encapsulated in processed PPP and cultured for various time points in vitro (Fig. 4A). The encapsulated DPSC and UC-MSC exposed high viability and proliferation levels at different experimental time points. The alamarBlue™ staining showed a 1.5 (*P* < 0.05), 3.1 (*P* ≤ 0.0001) and threefold change (*P* ≤ 0.0001) increase in the proliferation between DPSC + PPP vs PPP alone at day 3, day 7 and day 14, respectively. Also a1.8 fold change (*P* ≤ 0.0001)and 2.8 fold change (*P* ≤ 0.0001) increase were observed between UC-MSC + PPP vs PPP alone at day 7 and 14. (Fig. 4B).Histological sections (Fig. 4C) of UC-MSC or DPSC have revealed an increase in the cell number for DPSC and UC-MSC which coincides with the proliferation results. SEM images (Fig. 4D) revealed a porous structure of the PPP with random fibrin fiber spread around the biomaterial surface. Quantification of the porosity of PPP encapsulated with either DPSC or UC-MSC showed an increase in the size after 14 days of incubation compared to Day 1 (*P* ≤ 0.0001) (Fig. 4E).

### Quantification of the protein secretion and release profiles of PPP alone or after DPSC/UC-MSC Encapsulation

**Figure 5:**
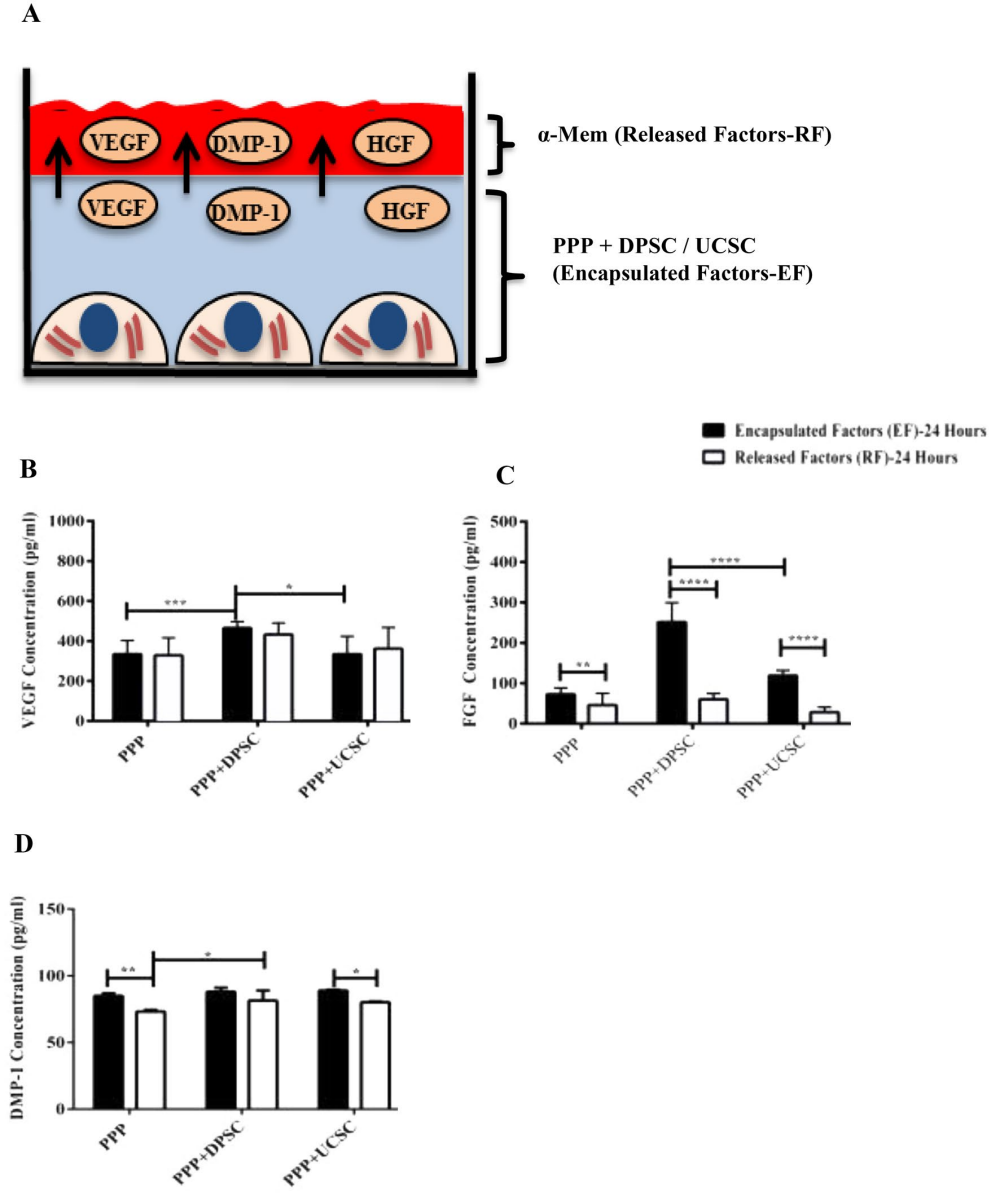
Quantification of protein factors released from PPP incubated with or without cells for 24 h. (**A**) Diagrammatic representation which shows how the different factors were release from PPP (Encapsulated Factors-EF) to the external α-MEM supernatant (Released Factors-RF). (**B**) Quantification of different proteins including VEGF, FGF and DMP-1 using an ELISA have demonstrated an increase in the expression of the encapsulated VEGF (EF) between PPP + DPSC and PPP + UC-MSC after 24 h of incubation (*P* ≤ 0.01). (**C**) Also, for FGF has observed an increase in the encapsulated FGF (EF) after 24 h between PPP + DPSC and PPP + UC-MSC (*P* ≤ 0.0001). For DMP-1, there was an increase in the encapsulated protein expression vs released for PPP after 24 h of incubation (*P* ≤ 0.01). All data are represented as a mean with the associated SEM (*n* = 3) of a minimal three donors.

The protein secretion or release profile of PPP with or without cells was performed in both the conditioned media (released factors, RF) or within the scaffold ( encapsulated factors, EF) by squeezing the scaffold followed by ELISA of different protein factors including VEGF, FGF, and DMP-1 as shown in Fig. 5A.The results showed a 1.3 fold increase (*P* ≤ 0.01) in the protein expression of the encapsulated VEGF between PPP + DPSC and PPP + UC-MSC after 24 h of incubation (Fig. 5B). Also, a 2.1-fold increase of (*P* ≤ 0.0001) increase in the protein expression of the encapsulated FGF (EF) after 24 h between PPP + DPSC and PPP + UC-MSC (Fig. 5C). Finally, for protein DMP-1 protein expression, no significant difference was observed in the protein expression between PPP + DPSC and PPP + UC-MSC encapsulated factors (EF) or released factors (RF) after 24 h of incubation.

### In vivo Implantation of dentin-disc/MSC-PPP constructs and Evaluation of dentin and angiogenic formation

**Figure 6:**
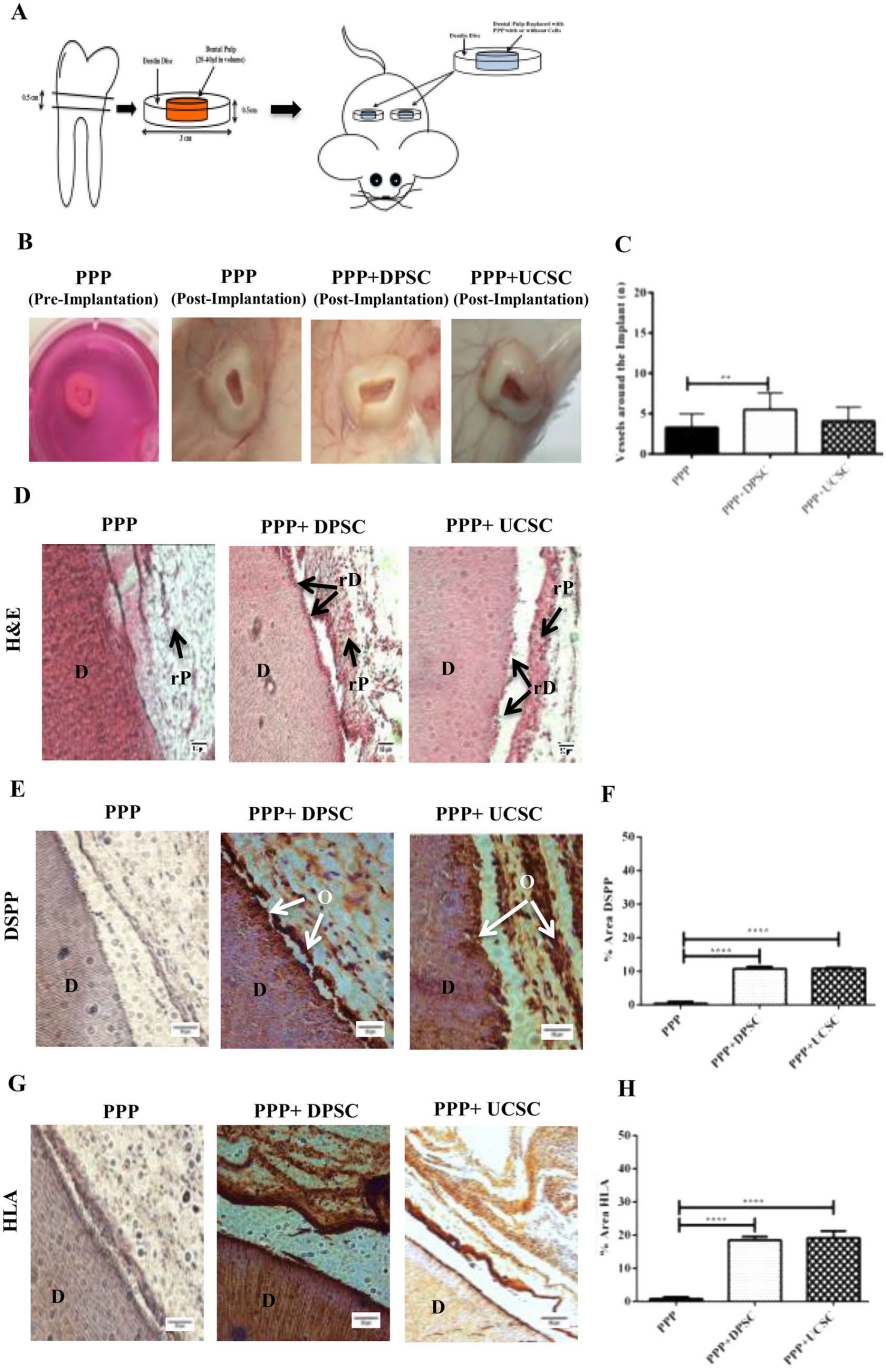
The Dentin/Disc scaffold model for dental pulp tissue engineering. (**A**) Schematic representation of the construct and its implantation in a mouse model. Dentin discs were sliced at a 0.5 cm diameter from extracted human molar teeth. The human pulp has been removed and instead the pulp cavity has been filled with PPP. Two dentin/PPP scaffolds were implanted subcutaneously on the dorsum of a mouse for a period of 30 days. In vivo evaluation of the vascularization of the Dentin/Disc PPP- DPSC and UC-MSC. In order to determine the angiogenic capacity between DPSC and UC-MSC the Dentin/Disc PPP model in NSG mice. The mice were divided into 3 different groups PPP (no cells), DPSC and UC-MSC. The different cells (1 × 10^6^) were mixed with PPP and implanted subcutaneously. (**B**) At 30 days post transplantation the implants were harvested, and images were taken and (**C**) Quantification of the vessels around the implant was performed using the image J software. The results have shown an increase in vessels formation around the dentin disc for the PPP + DPSC compared to PPP alone (*P* ≤ 0.01) (All data are represented as a mean with the associated SEM (*n* = 3) of a minimal three donors. Histological analysis of Dentin-Disc/PPP model after 30 days of implantation subcutaneously in a mouse (**D**) H&E staining of dentin/disc PPP with DPSC or UC-MSC has shown that cells were differentiated toward an odontoblast-type cell *D* Dentin Disc (Original Dentin), *rP* Regenerated Pulp-like tissue, *rD* Regenerated Dentin-like tissue. Arrows demonstrate that the DPSC or UC-MSC were migrated towards dentin disc and slightly entered in the dental tubule canal forming a slight line of new dentin-like tissue which is parallel to the original dentin. Immunohistochemical analysis of Dentin-Disc/PPP model after 30 days of implantation subcutaneously in a mouse. (E + G) Arrows indicate the DSPP staining of dentin/disc PPP with DPSC or UC-MSC have differentiated towards an odontoblast-type cell. HLA-A staining was used as positive control indicating the human origin of cells *O* odontoblast-type cell. (F + H) For the quantification of the amount of DSPP and HLA-A expression image J was used. The results have shown an increase for PPP + DPSC compared to PPP alone (*P* ≤ 0.0001) and also a difference for PPP + UC-MSC compared to PPP alone (*P* ≤ 0.0001) for both DSPP and HLA-A markers.

The different cell sources were encapsulated within the PPP and the dentin-discs/MSC-PPP constructs were implanted subcutaneously for 30 days (Fig. 6A). Images were taken of the implant’s prior removal (Fig. 6B) and an evaluation of the vessel formation around the dentin dentin-discs/PPP was performed with image J. The results showed a 1.6-fold increase (*P* ≤ 0.01) in vessels formation around the dentin-disc for the PPP + DPSC compared to PPP alone (Fig. 6C). After imaging, the dentin-discs/PPP scaffolds were removed, decalcified, and processed for histology. The hematoxylin and eosin staining (Fig. 6D) depict a high incorporation of the DPSC and UC-MSC in the PPP, with a migration profile towards the dentin wall forming a dense layer of new mineralized dentin-like tissue. Additionally, Van Gieson staining showed a matrix with collagen deposits (black arrows) in both PPP-DPSC and PPP-UCSC, probably from the cells differentiated to odontoblast-like phenotype (Supplemented Fig. 1C). Immunohistochemical staining revealed a positive staining for Dentin sialophosphoprotein (DSPP) which implies the formation of new dentin and odontoblast formation for both DPSC or UC-MSC, as presented in Fig. 6E. The quantification of DSPP expression by image J revealed a 28.1 fold change (*P* ≤ 0.0001)increase for PPP + DPSC compared to PPP alone and 28.5 fold change (*P* ≤ 0.0001) difference for PPP + UC-MSC compared to PPP (Fig. 6F). Human leukocyte antigen (HLA- A) was used to confirm the persistence of the encapsulated human cells after transplantation. As shown in Fig. 6G, most of the cells stained positively for the antibody confirming the human cell origin of these cells. The image analysis of the sections revealed an increase in the HLA-A expression for PPP + DPSC (24.6-fold change) and PPP + UC-MSC (25.5-fold change) (*P* ≤ 0.0001).

## Discussion

The main objective of this study was to investigate the efficacy of PPP as a cell delivery agent of allogenic or autologous MSC using a dentin/disc mouse model for dental pulp regeneration. Initially, we examined differentially expressed regulatory factors involved in key biological function of MSC isolated from the umbilical cord or dental pulp tissues. The characterization included comparisons of their proliferation potential, mesodermal differentiation, surface antigen expression and finally for their angiogenic potential, both in vitro and in vivo through a tubule and plug transplantation assays. While DPSC showed a slight proliferation and angiogenic advantage in vitro, UC-MSC displayed a higher migratory ability. It is very challenging to draw conclusions or extrapolate these results as the extent of donor variability throughout the characterization process can lead to high inconsistencies. Also, MSCs from the same source have shown significant differences that were associated to demographic or genetic variations. In contrast, the results of the in vivo plug assay have shown a significant increase in the hemoglobin content of DPSC and UC-MSC compared to the control (Matrigel alone), however, no differences were observed between DPSC and UC-MSC in the formation of new vessels around the implant. The difference between the angiogenic results obtained in the tubule versus the plug transplantation assays could be related to both experimental timing and microenvironment conditions. Since the main difference between the two sources where more prominent under hypoxic conditions, limb ischemia models might be more adequate to consider in the future for assessing angiogenic properties of MSCs. Taking these results into consideration, both cell sources represent good candidate for REP. While using DPSC might seem more logical for dental pulp regeneration, being a homologous tissue, the clinical translation of DPSC for dental pulp regeneration meets several obstacles such as a tooth needs to be removed to extract the DPSC (pulpectomy). However, it can still be considered where autologous use is desired. UC-MSCs offer an advantage as higher number of cells can be isolated from a single donation, making it more adequate for allogenic application. This is also representing a particular interest for reducing both batch to batch variation and costs. To achieve optimal dental pulp regeneration, beside selecting the most adequate cell source, the choice of an appropriate scaffold is necessary to achieve vascularization and formation of new dentin on the existing dentin surface. Previous dental pulp regeneration studies have used collagen membranes to deliver cells for dental regeneration, however, the results were discouraging mainly due to the contraction of the gel membranes were obstructing the regeneration of hard or soft tissue^45–47^. Synthetic membranes were also used previously such as PLG or poly-L-lactic acid are much more resistant to contraction than collagen, however the slow degradation rate hindered the pulp-like regeneration process^13^. Other sophisticated synthetic biomaterials showed interesting results in different regenerative medicine application, such as for heart regeneration. While the high-price can be justified for life-saving treatments it is difficult to vindicate them for dental repair. To overcome these drawbacks and challenges, Plasma Poor in Platelets (PPP) was used as a vehicle of choice to deliver MSCs for dental regeneration. PPP is a natural biomaterial with similar constitution to PRP (Plasma Rich in Platelets) but with less platelets. Beside its cell support properties, it also provides the third fundamental element for tissue engineering: the cytokines and growth factors. While other synthetic material can be supplemented with defined factors, it is very challenging and cost-ineffective to add all the proteins that are naturally present in PPP. Indeed, PPP contains many valuable growth factors such as VEGF, PDGF among others which potentially could help the formation of new dentin and vascularization of the new dental pulp. Also, PPP is not an osteoconductive biomaterial such as hydroxyapatite of tricalcium phosphate (a hard type biomaterial) which will convert all the dentin pulp area to an undesirable hard tissue. The final choice between PPP compared to PRP was also due to the high content of platelet levels that might have an adverse effect on DPSC, and proliferation and differentiation as shown previously. It seems that the PRP is only effective at specific platelets levels therefore for a dental pulp regeneration application might have been a limitation rather than advantage^39^. Finally, the choice of PPP from a universal donor had also a strategic translational application approach for allogenic therapies. PPP due to the low level of platelets compared to PRP could be refrigerated and shipped at longer distances whereas PRP always had to be extracted and delivered always fresh. This results in a significant reduction of manufacturing cost that makes the therapy more standardized and accessible. The suitability of PPP as a choice of scaffold was investigated macro-structurally with scanning electron microscopy (SEM) with or without cells. Interestingly, SEM images of PPP have shown that the fibrin fibers are randomly organized whereas the fibrin fibers were aligned when PPP was encapsulated with either UC-MSC or DPSC at day 14.This indicates a good compatibility and also a remodeling effect of the encapsulated cells within the scaffold. Additionally, the viability of DPSC or UC-MSC in vitro was investigated. The proliferation results demonstrated and that DPSC grow faster during a period of 14 days encapsulated in PPP in comparison to UC-MSC. Similar results have been observed in a different study^39^. Talking in consideration that PPP is a fibrin clot with many growth factors encapsulated, the release profile of VEGF, FGF and DMP-1 was quantified. There was an increase in the expression profile of all growth factors when cells were encapsulated in the PPP (internal) compared to PPP alone. The number of factors released in the culture media was the sum of the constitutive factors released by the PPP and the protein secreted de novo by the encapsulated stem cells through diffusion. To evaluate the interaction between the PPP, the encapsulated stem cells, and a dental cavity in vivo*,* a tooth-slice model was used to generate the Dentin/Disc-PPP-MSC construct. At 4 weeks post-transplantation, DPSC or UC-MSC encapsulated in PPP migrated from the PPP towards the open pores of the dentin wall. The newly deposited dentin-like tissue appeared to tightly adhere to the original dentin. The newly formed mineralized tissue also appeared to fill into the space of dentinal tubules. Dentin/disc-PPP with either UC-MSC or DPSC were differentiated towards a more hard-type tissue (odontogenic phenotype). The presence of human cells was confirmed with HLA-A staining. These odontoblast-like cells migrated towards the dentin were well organized and aligned as the natural counterparts as confirmed with the expression of DSPP. No significant difference was observed in DSPP expression between DPSC or UC-MSC. The chemotaxis might be due to growth factors such as the transforming growth factor that is located in the dentin and induces the migration of MSC from the PPP towards the dentin wall^48^. It has been shown that cultured human DPSC seeded onto treated dentin discs develop an odontoblast-like phenotype. Moreover, the cells presented a polygonal or columnar morphology that extended into the dentinal tubules, with a polarized organization of odontoblasts and displayed high levels of ALP activity^49^.This means that the induction of differentiation is initiated by the dentin disc that is supported or enhanced by differentiation-related factors present in PPP or secreted by MSC (DPSC or UC-MSC). These factors include: transforming growth factor-β1 (TGF-β1), insulin-like growth factor-1, bone morphogenetic protein and dentin matrix protein^35,37,50,51^. Apart from dentin formation, vascularization is another essential event for providing blood supply to the regenerated tissues. The Dentin-Disc implant was not sealed to allow a more effective capillary invasion inside the Dentin-Disc. Vessels were grown around the implant as seen in the images providing the appropriate nutrients to the encapsulated MSC allowing the cells to proliferate and then differentiate toward a more odontogenic phenotype. Future planed experiments involve the use of larger animals, such pigs or ferrets to regenerate these tissues in situ, within the jaw bone^52–54^. Recent findings from our clinical trial (registered at ClinicalTrials.gov (NCT03102879)) with human UC-MSCs encapsulated in PPP for REPs in mature teeth with apical lesions demonstrated an alternative method to recuperate vitality in mature teeth^55^.

## Conclusion

We demonstrate here, the feasibility of the use of PPP as cell delivery vehicle for DPSC or UC-MSC and an enhancer of the differentiation potential of MSC towards a more odontogenic phenotype. This study provides evidence of a manageable, cost-effective, xenofree scaffold that is compatible with either autologous or allogenic strategy for dental pulp regeneration. This attempt if successfully implemented could make accessible REPs treatment to a large part of the world, contributing in improving global health conditions.

## Supplementary Information

Supplementary Legends.

Supplementary Information 2.
