# Supplementary material for: Delivery of affordable and scalable encapsulated allogenic/autologous mesenchymal stem cells in coagulated platelet poor plasma for dental pulp regeneration
Source: Sci Rep. 2022 Jan 10;12:435. doi: 10.1038/s41598-021-02118-0 (PMC8748942; doi:10.1038/s41598-021-02118-0)
Supplement: Supplementary file 1 — Supplementary Legends. [file 41598_2021_2118_MOESM1_ESM.docx]

**Supplementary Figure 1. DPSC and UC-MSC express the common mesenchymal stem cell markers.** (A)MSCs were stained with labeled monoclonal antibodies against known MSC surface markers (red) and their respective isotypes (grey), cells were analyzed by flow cytometry. All MSCs were positive for CD105, CD90, CD73 and negative for CD34, CD45, CD19 and HLA-DR. **(B)DPSC and UC-MSC display similar mesodermal differentiation potential.** Images of MSC differentiation to a three-lineage following incubation with differentiation medium for 30 days and stained with Oil red O (adipocytes), Alzarin red (osteocytes) and Safranin O (Chondrocytes). (C) Van Gieson staining showed a matrix with collagen deposits (black arrows) in both PPP-DPSC and PPP-UCSC, probably from the cells differentiated to odontoblast-like phenotype
