# Supplementary figures and images for: Delivery of affordable and scalable encapsulated allogenic/autologous mesenchymal stem cells in coagulated platelet poor plasma for dental pulp regeneration
Source: Sci Rep. 2022 Jan 10;12:435. doi: 10.1038/s41598-021-02118-0 (PMC8748942; doi:10.1038/s41598-021-02118-0)

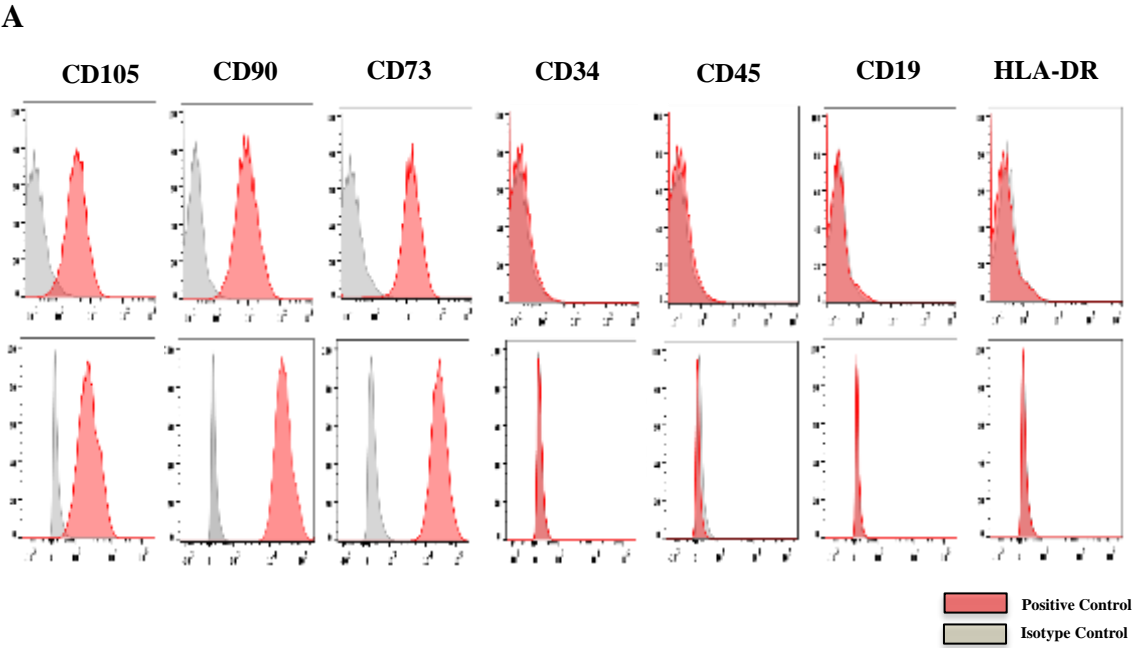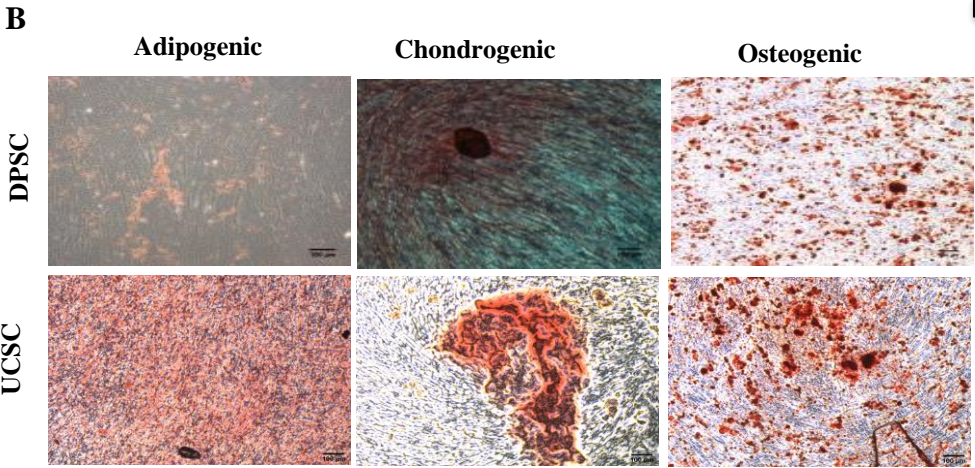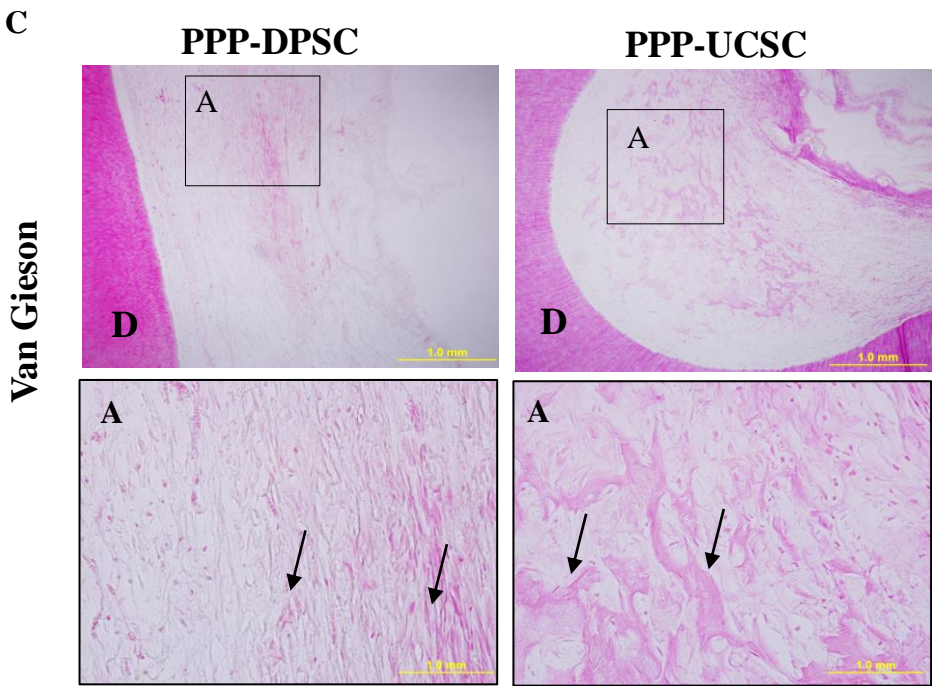

Supplementary Figure 1

Supplement: Supplementary file 2 — Supplementary Information 2. [file 41598_2021_2118_MOESM2_ESM.pdf]
